# Short- and long-term effects of social hierarchy level on seminal quality and behavior traits in goat bucks

**DOI:** 10.1007/s11250-025-04814-9

**Published:** 2025-12-19

**Authors:** José R. Haro-Torres, Gustavo Ramírez-Valverde, Saúl Hernández-Aquino, Rodrigo Flores-Garivay, Lorenzo Buenabad-Carrasco, Juan González-Maldonado

**Affiliations:** 1 Instituto de Ciencias Agrícolas, Universidad Autónoma de Baja California, Ejido Nuevo León, Mexicali, 21705 México; 2 Colegio de Postgraduados, Departamento de Estadística, Montecillo, Texcoco 56230 México; 3 Facultad de Zootecnia y Ecología, Universidad Autónoma de Chihuahua, Chihuahua, 31453 México

**Keywords:** Dominant, Sexual, Social, Subordinate, Sperm

## Abstract

Social hierarchy affects semen quality and social interactions in males. However, the long-term effects of social hierarchy on semen quality, sexual, and social traits in goat bucks are unknown. Goat bucks were assigned to the dominant and subordinate groups. Semen quality, sexual, and social behavior traits were evaluated every other day for six days to establish a short-term effect, and every week for 25 weeks to establish a long-term effect of social hierarchy. There was not a short-term effect (*P* > 0.05) of social hierarchy in social and sexual behavior traits, but dominant males produced seminal samples with higher (*P* < 0.05) progressive motility than subordinate males. There was a long-term effect on social and sexual behavior, but no changes were related to semen quality traits. The subordinate males received more hits during feeding time (*P* < 0.05), and they performed a lower number of vocalizations than dominant males (*P* < 0.05). In conclusion, social hierarchy affects the semen quality in the short-term, and social and sexual behavior traits in the long-term.

## Introduction

The effects of social hierarchy level (dominant vs. subordinate) on seminal quality and behavioral traits in male ruminants have been investigated over the past years (Ekiz et al. 2024; Shreffler and Hohenboken 1974). However, it is still a trending topic because of the existence of contradictory results, and there are also questions that remain to be answered. Regarding semen quality, some authors agree that dominant rams produce ejaculates of higher sperm concentration and volume than subordinate rams (Sifuentes-Lamónt et al. 2022; Véliz-Deras et al. 2022), while other authors have reported no effect of social hierarchy level in those and other semen quality traits on rams and goat bucks (Vázquez et al. 2012; Sánchez-Dávila et al. 2018).

In addition, most of the available scientific literature studied the effects of social hierarchy on semen quality, sexual and social behavior in rams and bucks for a shorts period of time ( 2 to 63 days); (Sánchez-Dávila et al. 2018; Ungerfeld et al. 2019, 2023; Eegül-Ekiz et al. 2020; Sifuentes-Lamónt et al. 2022). To our knowledge, only one study has explored the long-term effects of social hierarchy on semen quality and sexual behavior in rams (Aguirre et al. 2007). Therefore, it awaits to be clarified if the known effects of social hierarchy on semen quality and behavioral traits are controlled by time length in goat bucks. The objective of the present study was to establish the short and long-term effects of social hierarchy on seminal quality, sexual and social behavioral traits in male goats.

## Materials and methods

### Location and experimental units and design

The study was carried out from mid-April to mid-November in a goat farm located at the Ejido Nuevo León, Mexicali, México (32°25’01.1"N 115°11’10.3"W). The climate of the region is classified as a hot desert (Bwh); the highest and lowest temperatures recorded during the summer and winter seasons are 50 °C and − 5 °C, respectively, and the average annual rainfall is 88 mm (Garcia, 2004). The breeding season lasts from May to February. The experimental units (10 bucks, 2.13 ± 0.17 years old, 1 Boer and 9 Boer × Kalahari red breed, 26.92 ± 2.90 cm of scrotal circumference and 66.80 ± 3.22 kg of body weight) were allocated in individual pens for at least eight months previous to the start of the study. At day 0 of the study, the goat bucks were fasted for at least 16 h, and they were then placed at the same time in a single pen (5.3 × 10.3 m). Alfalfa hay was provided to the bucks in five individual feeders (the space in each feeder allows access to only one buck at the time). The bucks that ate from the same feeder for at least 1 min were assigned to the dominant group, the rest of the bucks were assigned to the subordinate group (Synnott and Fulkerson 1984). The morphological traits of the experimental units in the experimental groups are shown in Table 1. The experimental period lasted seven months.

**Table 1:** Morphological traits of goat bucks in the dominant and the subordinate groups

Morphological trait*	Group	
	Dominant (*n* = 5)	Subordinate (*n* = 5)
Body weight (kg)	66.60 ± 3.04	67.0 ± 3.74
Scrotal circumference (cm)	27.60 ± 2.91	26.90 ± 3.22
Body length (cm)	137.40 ± 7.30	132.80 ± 6.94
Height (cm)	82.20 ± 1.30	81.70 ± 1.09
Thoracic perimeter (cm)	98.70 ± 4.05	93.0 ± 22.99
Space between eyes (cm)	16.10 ± 1.34	15.60 ± 1.08
Horn size	36.43 ± 6.08	38.40 ± 5.82

*Measurements were taken using a flexible plastic tape as previously describe elsewhere (Sifuentes-Lamónt et al. 2022): body length (length from the base of the tail and the snout), height (distance between the base of the foot and the whithers), horn size (average distance from the base to the tip of both horns)

### Animal feeding and housing

The goat bucks were housed in a single pen (5.3 × 10.3 m with a shade area of 23.64 m^2^). They were fed with alfalfa hay (1600 g^− 1^ buck^− 1^ d^− 1^) at 08:00 (800 g) and 17:00 (800 g) h, and they had ad libitum access to water.

### Semen sample collection and body measurements

The body weight was weekly recorded. The goat bucks were subjected to semen analysis and scrotal circumference measurement every other day from day cero (first day of grouping) to six days after grouping to detect a short-term effect of the social status (dominant vs. subordinate), and then every week for approximately seven months to stablish a long-term effect of the social status. The measurement of the response variables was occasionally suspended during the long-term period due to hostile weather conditions and technical inconveniences, but the males were sampled for a total of 25 weeks within a seven-months period.

The semen samples were collected with an artificial vagina, using a restrained non-in estrus goat. The reaction time was measured with a chromometer to determine the time lapse between buck release from a fixed point to ejaculation. The ejaculated volume was determined by weighing (Mocé et al. 2022). A sample of 5.0 µL of semen was diluted in water (1:400) to determine sperm concentration using a Neubauer chamber (Gore et al. 2020).

A 5.0 µL semen sample was diluted (1:200) in semen extender (Triladyl^®^: 60% water, 20% egg yolk, and 20% triladyl). The diluted semen sample was placed in a warm bath (28.0 °C) to further analysis. A sample of 10 µL of diluted semen was placed on a warm slide covered with a coverslip (22 × 22 mm) to assess the number of motile sperm and the number of spermatic cells with progressive motility, using a 40× magnification microscope objective (Velab- B4 microscope); each counting was made separately.

The number of normal and live/dead sperms was determined by smearing equal volumes of the diluted semen sample with eosin-nigrosin stain. The stained-fixed sample was examined using a 100× magnification microscope objective in oil immersion (Gore et al. 2020). A sample of 17 µL of diluted semen was mixed with 69 µL of 0.4% NaCl and 14 µL of eosin-negrosin (Padrik et al. 2012). A sample of 10 µL of the mix was placed on a slide covered with a coverslip (22 × 22 mm) to assess the number of sperm with a coiled tail, using 40× magnification microscope objective. A total of 100 sperm were counted for each semen quality determination.

### Social behavior traits before and after eating

Social interaction traits in dominant and subordinate goat bucks were recorded for 12 min before feeding time and for 12 min during feeding time. A group of five trained technicians recorded the number of times that every male buck hits (hit given), chases (chase given) mounts attempts (mount given) or it was hit (hit received), chased (chase received) and mounted attempt (mount received) by another male. In addition, the number of threats, fights, flehmen reflexes, vocalizations, and micturitions were recorded. To perform the social behavior traits evaluation, two goat bucks were assigned to each technician. The measurements were carried out before semen collections.

### Sexual activity of the bucks

To evaluate the sexual activity of the males, physical contact between one buck at the time with 15 goats, located in a 5.3 × 10.3 m pen, was allowed for three minutes after semen collection. The number of mounts, mount attempts, ano-genital sniffing, vocalization, and penne exteriorization were counted after each buck made contact with the goats. In addition, the time that each buck spent in other activities not related to sexual behavior (eating or exploring) was recorded with a chronometer. The measurements were carried out immediately after semen collection.

### Statistical analysis

To accurately compare the two treatments and maximize the use of the sample size, the response variables were analyzed using mixed general linear models and generalized linear mixed models, considering repeated measures over time when applicable. The continuous variables were analyzed using general linear models and mixed linear models for repeated measures. In this case, different correlation structures were evaluated, selecting the one that provided the best fit according to the Akaike Information Criterion (AIC) and Bayesian Information Criterion (BIC). When significant differences were detected, mean separation was performed using Tukey’s test. The dichotomous categorical variables were analyzed using generalized linear mixed models with a logit link function. The count categorical variables were analyzed using generalized linear mixed models with a log link function. The analyses were performed using Infostat, integrating statistical packages from R (version 3.6.3). In the mixed models, the maximum likelihood estimation (REML) was applied to the analysis, which is robust to unbalanced data and allows the inclusion of all available observations without biasing parameter estimates. This approach assumes that the missing data were missing at random (MAR), which is reasonable in the present study because data loss was exclusively due to external factors unrelated to the experimental design. Variables where the statistical model did not converge were removed from the result section.

## Results

A short-term effect of social hierarchy level (dominant vs. subordinate) was observed in seminal quality, and a long-term effect was observed in seminal quality and sexual behavior traits. The social behavior traits were not affected by either the short- or the long-term hierarchy level in goat bucks (Table 2). The live weight was not affected (*P* > 0.05) by social status (71.43 ± 1.12 vs. 68.81 ± 1.12 kg for dominant and subordinate groups).

**Table 2:** Short and long-term effects of social hierarchy level on seminal quality and behavioral traits in goat bucks

Parameter		Social hierarchy level			*P*-value
		Dominant	Subordinate		
**Short term effects on semen quality traits**					
Scrotal circumference (cm)	26.98 ± 0.93		28.51 ± 0.93		0.2784
Volume (mL)	0.70 ± 0.17		0.47 ± 0.17		0.3701
Sperm concentration (×10^6^ mL^− 1^)	3760.0 ± 436.51		2492.0 ± 436.51		0.0740
Motile sperm (%)	90.0 ± 1.0		87.0 ± 2.0		0.2445
Sperm with progressive motility (%)	99.00 ± 0.26		98.0 ± 1.0		0.0130
Membrane integrity (%)	22.00 ± 3.0		19.0 ± 3.0		0.4118
Normal sperms (%)	97.0 ± 1.0		97.0 ± 1.0		0.9844
Live sperms (%)	97.0 ± 1.0		96.0 ± 1.0		0.4760
Reaction time (s)	11.94 ± 2.51		17.42 ± 2.51		0.1609
**Short-term effects on sexual behavior traits**					
Flehmen reflex	0.37 ± 0.21		0.76 ± 0.34		0.3212
Penne extrusion	2.3E-4 ± 1.70		5.9E-9 ± 0.06		0.9999
Vocalization	1.10 ± 0.56		1.65 ± 0.76		0.5557
Sniffing	11.28 ± 1.96		10.24 ± 1.84		0.7004
Time exploring	88.37 ± 13.24		83.83 ± 13.24		0.8175
**Shor-term effects on social behavior traits** *					
Hit given-BF	1.29 ± 0.36		0.60 ± 0.22		0.1007
Hit given-DF	1.2E-7 ± 7.1E-5		0.50 ± 0.21		0.9798
Hit received-BF	1.43 ± 0.50		0.88 ± 0.33		0.3523
Hit received-DF	3.0E-5 ± 0.09		3.3E-3 ± 6.62		0.9990
Threats-BF	0.58 ± 0.27		0.48 ± 0.24		0.1777
Fights-BF	2.19 ± 0.83		1.02 ± 0.42		0.1470
Fights-DF	6.7E-8 ± 5.1E-4		7.3E-6 ± 0.10		0.9998
Mount attempt received-BF	2.4E-5 ± 0.08		2.8E-3 ± 3.31		0.9989
Flemen reflex-BF	6.9E-9 ± 0.05		2.6E-11 ± 2.0E-4		0.9999
Vocalization-BF	1.69 ± 1.18		0.02 ± 1.06		0.9429
Vocalization-DF	0.01 ± 6.81		0.01 ± 12.98		0.9998
Micturition-BF	8.2E-4 ± 0.17		2.1E-6 ± 1.4E-3		0.9939
**Long-term effects on semen quality traits**					
Scrotal circumference (cm)	27.16 ± 0.84		29.1 ± 0.84		0.1305
Volume (mL)	0.69 ± 0.07		0.65 ± 0.07		0.6423
Sperm concentration (×10^9^ mL^− 1^)	3517.14 ± 289.00		3372.00 ± 289.00		0.7261
Motile sperm (%)	86.60 ± 1.28		87.10 ± 1.25		0.4499
Sperm with progressive motility (%)	97.70 ± 0.39		98.50 ± 0.27		0.1058
Membrane integrity (%)	16.20 ± 0.80		18.40 ± 0.88		0.3599
Normal sperms (%)	96.71 ± 0.19		96.15 ± 0.216		0.1832
Live sperms (%)	95.20 ± 0.40		95.80 ± 3.76		0.1905
Reaction time (s)	16.60 ± 1.92		14.70 ± 1.92		0.5047
**Long-term effects on sexual behavior traits**					
Flehmen reflex	0.91 ± 0.18		1.09 ± 0.21		0.8787
Penne exteriorization	0.04 ± 36.60		0.03 ± 29.70		0.3872
Vocalization	2.36 ± 0.28		1.45 ± 0.19		0.01800
Sniffing	11.20 ± 0.59		12.20 ± 0.63		0.5886
Time exploring	49.70 ± 4.73		49.60 ± 4.74		0.9566
**Long-term effects on social behavior traits** *					
Hit given-DF	3.20E-05 ± 0.11		4.12E-05 ± 7.31		0.4800
Hit received-BF	1.02E-07 ± 0.59E-3		2.66E-07 ± 1.5E-3		0.1286
Hit received-DF	0.18 ± 0.04		0.31 ± 0.06		0.0444
Threats-BF	2.19E-11 ± 8.22E-07		1.37E-12 ± 2.51E-07		0.4757
Fights-BF	3.83E-6 ± 0.03		7.06E-6 ± 0.01		0.1154
Fights-DF	0.006 ± 8.31		0.004 ± 5.75		0.4342
Vocalization-BF	1.85 ± 0.71		3.13 ± 1.19		0.4516
Vocalization-DF	0.002 ± 9.3		0.0007 ± 41.2		0.7497

^*^BF: before feeding; DF: during feeding

## Discussion

Authors have reported that dominant rams produce semen samples of higher concentration and volume than subordinate males (Aguirre et al. 2007). However, other authors have reported no effects (Vázquez et al. 2012; Sánchez-Dávila et al. 2018) or limited effects (Sifuentes-Lamónt et al. 2022; Véliz-Deras et al. 2022) of hierarchy level on those variables in rams and goat bucks. Disagreement among research studies might be related to the number of ejaculate collections per male to perform semen analysis, which vary from two (Sifuentes-Lamónt et al. 2022; Véliz-Deras et al. 2022), five (Vázquez et al. 2012), nine (Sánchez-Dávila et al. 2018), and 24 (Aguirre et al. 2007). To increase results accuracy, it is advisable to increase the number of samples during seme analysis, because of the high variability of semen traits values (Giriboni et al. 2015). To our knowledge, there is no specification regarding the minimum number of ejaculate samples required to obtain accurate results about semen quality in goat bucks. However, there is a recommendation in bulls, which states that a minimum of five semen samples collected within two weeks is necessary to properly rate the ejaculate fertility (Swanson and Herman 1941).

In the present study, the progressive motility was superior in dominant than in subordinate bucks only during the short-term effect of social hierarchy level. However, after the long-term analysis, there was no effect of the social hierarchy status on any of the measured variables in semen samples. It is likely that during the short-term period, the grouping of goat bucks caused a physiological stress (Sánchez-Dávila et al. 2018) that temporarily disrupted the sperm motility in subordinate males (Juárez-Rojas et al. 2017). The lack of effects of the hierarchy level during the long-term might be attributed to physiological adaptations to such stress.

In order to observe agonist behavior between dominant and subordinate males, a competition state must be established either by space limitations, feed and female availability (Ortíz et al. 2001). For example, authors have provoked a competition state to stablish a hierarchy level by exposing males to an estrus ewe within a confine space (6.25 m^2^) (Sifuentes-Lamónt et al. 2022). In the present study, there was no space, food a female availability limitation to elicit competition. However, the subordinate males received more hits during feeding time, which clearly signals their low social status. Regardless, no physical injuries were recorded during the entire study. In agreement, research has shown that the welfare of the low rank males is not compromised by the presence of dominant males as long as there are no feed and space limitations (Eegül-Ekiz et al. 2020).

The number of vocalizations was the only variable related to sexual behavior that was affected by social hierarchy. The dominant goat bucks performed a higher number of vocalizations than subordinate males. This difference might be explained by a possibly higher libido of dominant than subordinate males (Aguirre et al. 2007).

The results of the current study demonstrate that in the short-term, the hierarchy level does not affect the sexual and social behavior traits, but the dominant males produce semen samples with higher progressive motility than the subordinate males. In the long-term, hierarchy level does not affect semen quality, but subordinate males perform a lower number of vocalizations, and they receive more hits during feeding time than the dominant males. However, further research evaluating the effects of social hierarchy on semen quality, social and sexual behavior traits must overcome the limitations of the present study, such as the small sample size.

## Data Availability

The raw data that support the research is available upon request to the corresponding author.
